# An Interpretable Machine Learning Model to Predict Cortical Atrophy in Multiple Sclerosis

**DOI:** 10.3390/brainsci13020198

**Published:** 2023-01-24

**Authors:** Allegra Conti, Constantina Andrada Treaba, Ambica Mehndiratta, Valeria Teresa Barletta, Caterina Mainero, Nicola Toschi

**Affiliations:** 1 Department of Biomedicine and Prevention, University of Rome ‘Tor Vergata’, Via Montpellier 1, 00133 Rome, Italy; 2 Massachusetts General Hospital, Boston, MA 02114, USA; 3 A. A. Martinos Center for Biomedical Imaging, Boston, MA 02129, USA

**Keywords:** cortical atrophy, multiple sclerosis, machine learning, explainability, rim lesions, leukocortical lesions

## Abstract

To date, the relationship between central hallmarks of multiple sclerosis (MS), such as white matter (WM)/cortical demyelinated lesions and cortical gray matter atrophy, remains unclear. We investigated the interplay between cortical atrophy and individual lesion-type patterns that have recently emerged as new radiological markers of MS disease progression. We employed a machine learning model to predict mean cortical thinning in whole-brain and single hemispheres in 150 cortical regions using demographic and lesion-related characteristics, evaluated via an ultrahigh field (7 Tesla) MRI. We found that (i) volume and rimless (i.e., without a “rim” of iron-laden immune cells) WM lesions, patient age, and volume of intracortical lesions have the most predictive power; (ii) WM lesions are more important for prediction when their load is small, while cortical lesion load becomes more important as it increases; (iii) WM lesions play a greater role in the progression of atrophy during the latest stages of the disease. Our results highlight the intricacy of MS pathology across the whole brain. In turn, this calls for multivariate statistical analyses and mechanistic modeling techniques to understand the etiopathogenesis of lesions.

## 1. Introduction

Multiple sclerosis (MS) is a chronic, immune-mediated disease of the central nervous system and the leading cause of nontraumatic neurological disability in young adults in the Western world [1]. Classically considered a white matter disease, MS also involves gray matter (GM) and is characterized by focal and/or diffuse inflammation, demyelination and neurodegeneration. GM neurodegeneration is now recognized as a crucial component of disease pathology and a substrate of clinical disability [2]. Moreover, the gray matter volumetric changes that occur during the disease are also relevant for monitoring treatment efficacy in clinical trials [3]. However, the mechanisms underlying the loss of GM in MS remain largely undetermined [4]. For example, it is unknown whether cortical tissue loss, the primary determinant of gray matter loss [5], is due to disconnection mechanisms caused by white matter (WM) lesions [6], local demyelination [7] or a combination or interaction of both. Using the increased sensitivity of ultrahigh field (UHF, 7T) magnetic resonance imaging (MRI) to identify cortical lesions in MS [8,9], it has been shown that WM lesions play a greater role than cortical lesions in cortical neurodegeneration [10]. However, thus far, no studies have compared the different destructive potential of different types of lesions in inducing cortical tissue loss. Among the different types of pathological lesions, chronic active lesions are of particular interest due to their association with the failure of repair mechanisms, which leads to progressive tissue destruction. Their hallmark, i.e., a paramagnetic rim, can be clearly identified in a high-resolution susceptibility-weighted MRI. Commonly called ‘rim lesions’, these findings have been observed in all stages of the disease, with a predominant localization in WM [11,12]. Nevertheless, the cortical location of rim lesions is possible, according to the observation of a paramagnetic rim in some leukocortical lesions [9,13]. Chronic active rim lesions are characterized by chronic inflammation, slow expansion, remyelination failure, and axonal loss. They have been associated with an overall higher lesion load, subcortical atrophy, ventricular enlargement, and more severe clinical disability [11,14,15]. Furthermore, the disruption of the structural network caused by chronic active lesions appears to be greater than that generated by other types of lesions [16].

In this study, by combining 7T MRI and machine learning algorithms based on extreme gradient boosting techniques and explainability strategies that uniquely identify the importance of each type of lesion (as well as other data) in each prediction, we aimed to assess the relationship and individual importance of different subtypes of cortical and WM lesions on cortical tissue loss. The flexibility and efficacy of modern gradient boosting algorithms allows us to maximize prediction power, while a machine learning approach, which includes train/test splits and hyperparameter optimizations, results in findings that are more generalizable than those of conventional statistical approaches. Furthermore, our objective was to also investigate whether the relationship between different subtypes of lesions and cortical neurodegeneration changes as a function of the disease stage.

## 2. Materials and Methods

### 2.1. Patient Population

**Figure 1:**
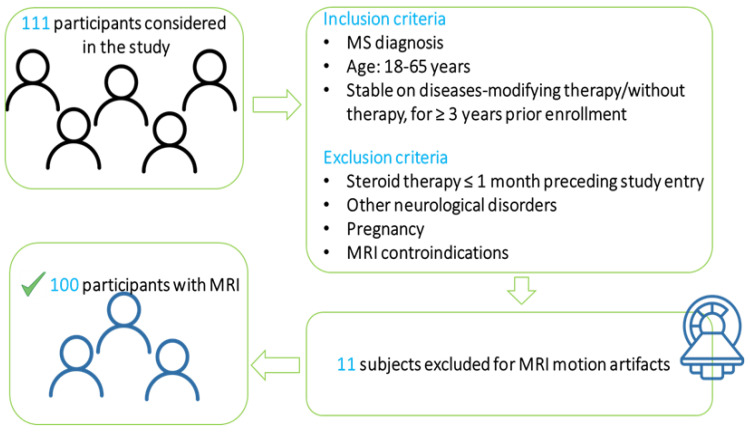
Study inclusion criteria.

One hundred and eleven patients were prospectively recruited at Massachusetts General Hospital (Boston). We included 74 patients with relapsing–remitting (RR) MS and 37 with secondary progressive (SP) MS. Figure 1 depicts the inclusion criteria. All protocols were approved by the institutional review board. Each participant gave written informed consent.

### 2.2. MRI Protocol

The assessment of WM and cortical lesions, as well as a paramagnetic rim surrounding the lesions, was performed on bidimensional T2*-weighted (T2*-w) MR images acquired at 7T (fast low-angle shot FLASH sequence, TR = 1700 ms, TE = 21.8 ms, resolution (x, y, z) = (0.33, 0.3, 1) mm^3^ resolution). Additionally, three-dimensional T1-weighted (3D T1-w) MR images were acquired at 3T (TR = 2530 ms, isotropic resolution of TE = 1200 ms, 0.9 mm^3^) for cortical thickness evaluation, as described below.

### 2.3. Image Processing

Cortical thickness was evaluated by segmenting 3D T1-w through the FreeSurfer (Software version 5.3.0, 2013, http://surfer.nmr.mgh.harvard.edu (accessed on 24 June 2021)) reconstruction stream. After vertexwise reconstruction of the pial and white surfaces, cortical thickness (evaluated as the distance between two corresponding vertices on the pial and white surfaces) was aggregated in a region of interest (ROI)-wise manner by averaging within-ROI values (Destrieux atlas [17]). ROI-wise values were then averaged to obtain the mean thickness in the left and right hemispheres and in the whole brain.

### 2.4. Lesion Identification

**Figure 2:**
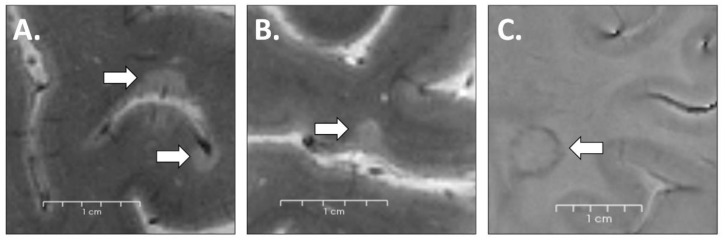
Examples of lesions visible on T2*-w images in a 48-year-old female MS patient (intracortical (**A**) and leukocortical (**B**) lesions identifiable in magnitude imaging; (**C**) rim lesion visible in phase-contrast imaging.

Lesions were identified via consensus between one radiologist and one neurologist (CAT, CM), each with approximately 20 years of experience in neuroimaging analysis, and segmented using Slicer (version 4.4.0; http://www.slicer.org, (accessed on 24 June 2021)). Cortical hyperintensities extending for at least three voxels across two consecutive slices on magnitude T2*-w image lesions were classified as (i) intracortical lesions if subpial or confined to the cortex (Figure 2A) and (ii) leukocortical lesions when they also involved the WM (Figure 2B).

All lesions presenting a hypointense peripheral margin on phase T2*-w images [18] (Figure 2C) surrounding an isointense to extralesional center were considered “rim lesions.”

Lesion counts and volumes were quantified using FreeSurfer and FSL (version 5.0).

### 2.5. Predictive Model

We employed prediction models for local and mean cortical thickness based on the extreme gradient boosting (XGBoost) technique [19]. Our models used 13 demographic variables (sex, patient age and age at disease onset) and lesion characteristics (rim lesion presence/absence; rim lesion load (dichotomous variable, <4 rim lesions ≥ 4 rim lesions [11]); count and volumes of rim, rimless, leukocortical and intracortical lesions). The model-building procedure was as follows:

(i) We randomly divided the data set in a stratified manner into training and test sets (70% and 30%, respectively).
(ii) We used a grid search (5-fold cross-validation) during training to optimize model hyperparameters (maximum depth of a tree; step size shrinkage used in update to prevent overfitting; the minimum sum of instance weight needed in a child).
(iii) We evaluated model performances by calculating, through the same Python script, the Pearson correlation (r) and *p* value between the real and predicted values in the test set.
(iv) The individual and cumulative contribution of each feature to the final prediction was assessed by calculating the Shapley additive explanations (SHAP) values [20].
(v) By repeating 50 times all the procedure on different 70/30 randomly split training and test sets, it was possible to obtain a confidence interval for both r- and *p*-values, as well as average SHAP values across repetitions.

All calculations were performed on a dual Xeon Workstation that was also connected to large-scale cloud computing resources [21]. To examine the relationship between lesion characteristics and brain atrophy at different stages of MS, we used the above procedure to predict mean atrophy (evaluated in the whole brain and both hemispheres) in two subject groups, G1 and G2. G1: 54 patients with MS from more than five years—age = 47 ± 8, age at onset = 30 ± 10; F:16, M:38; G2: 46 patients affected by MS from less than five years—age = 37 ± 8, age at onset = 35 ± 4; F:8, M:38. In this case, we employed a leave-one-out cross-validation (with hyperparameter optimization in a 5-fold cross-validation fashion on each training set) and assessed model performance in terms of both r and *p* values calculated on the observed and predicted values (54 for G1 and 46 for G2). All the above analyses were carried out in Python 3.6 and the scikit-learn module [22].

## 3. Results

**Figure 3:**
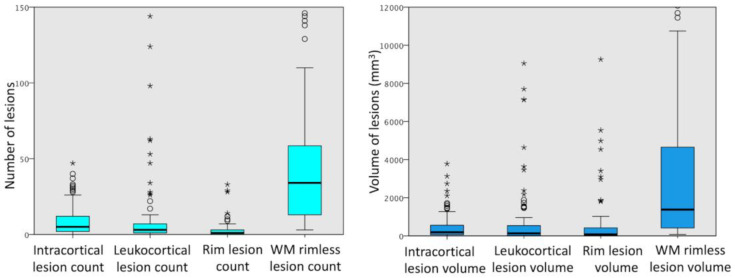
Boxplots summarizing the distribution of the number and volume of different types of cortical and white matter lesions from 100 multiple sclerosis patients. Boxplots represent the median, interquartile range and range. Points outside the whiskers are considered outliers (circles—cases with values between 1.5 and 3 box lengths from the nearer edge of the box; stars—cases lying more than 3 box lengths).

We evaluated 100 MS patients (74 RRMS and 26 SPMS; 76 female, 24 male; age = 43 ± 10, age at disease onset = 33 ± 9). The number and volume of different types of cortical and WM lesions evaluated using MRI are summarized in Figure 3. Circles and stars represent outliers with values between 1.5 and 3 box lengths from the nearest box edge and larger than 3 box lengths, respectively. In our cohort, all but four participants had at least one cortical lesion, while rim lesions were present in 63% of MS patients. When looking at lesion types, a susceptibility rim was visible in 4.5% (233/5161) of WM lesions and in 0.7% (14/1950) of cortical lesions (all leukocortical).

Table 1 shows the satisfactory performance of the model (mean *p* < 0.02 and mean Pearson r > 0.4 evaluated) when predicting mean cortical thickness, both in individual hemispheres and in the whole brain. This finding confirms a strong relationship between lesion characteristics and cortical atrophy, which is well-generalized to the test set.

**Figure 4:**
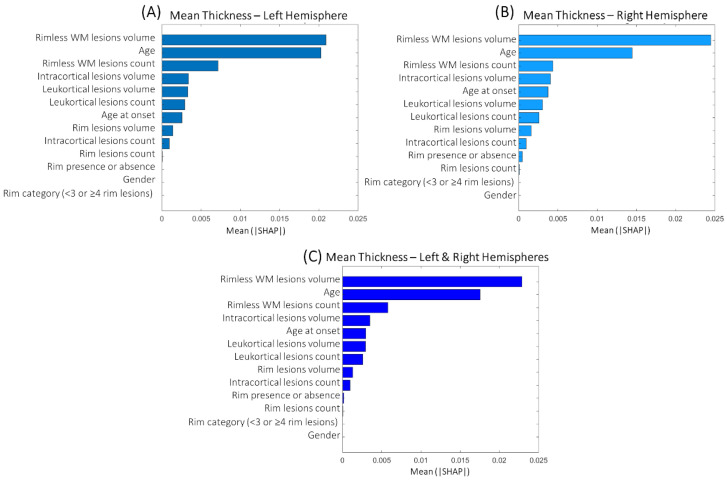
Mean feature importance evaluated across 50 repetitions obtained when predicting cortical thickness in single hemispheres (**A**,**B**) and whole brain (**C**).

Figure 4A–C show the feature importance ranking when predicting mean thickness values in individual hemispheres/entire brain (the top variables contribute more to the model than the bottom). The four most important characteristics for all these predictions were rimless WM lesion volume, patient age, rimless WM lesion count, and intracortical lesion volume.

**Figure 5:**
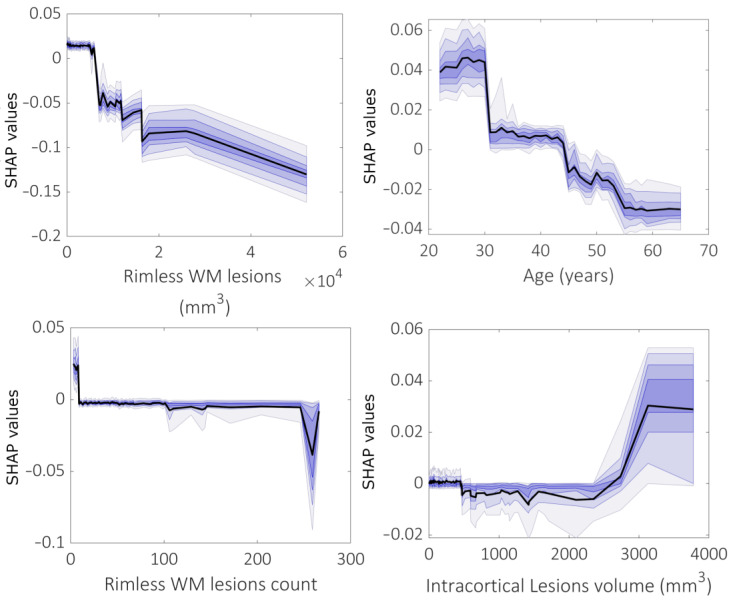
SHAP dependence plots of the rimless WM lesion volume, patient age, rimless WM lesion count and intracortical lesion volume obtained when predicting mean cortical thickness in the whole brain.

Figure 5 shows SHAP dependence plots, i.e., the impact of each feature on prediction performance as a function of the feature value itself when predicting globally averaged cortical thickness. Firstly, a decreasing dependence of importance on feature values is observed with the increased volume of rimless WM lesion load (i.e., volume and count), indicating that the smaller the lesion burden, the greater its role in predicting atrophy. SHAP values also decrease with increasing patient age, indicating a decrease in the contribution of this feature in predicting cortical thinning as its value increases. In addition, the volume of intracortical lesions becomes more important in the prediction of atrophy as its value increases.

When the analysis was repeated region by region, good prediction performances (which were termed “good” when they corresponded to statistically significant Pearson correlations with a value of <0.05 between real and predicted values) were obtained in 15 regions of the brain in both brain hemispheres (7 in the left hemisphere and 8 in the right hemisphere), as shown in Table 2. These regions mainly belong to the frontal and temporal cortices of the brain, with only the areas of the superior frontal gyrus (F1), the medial occipitotemporal and lingual sulcus, and the superior temporal sulcus found between the cerebral hemispheres.

**Figure 6:**
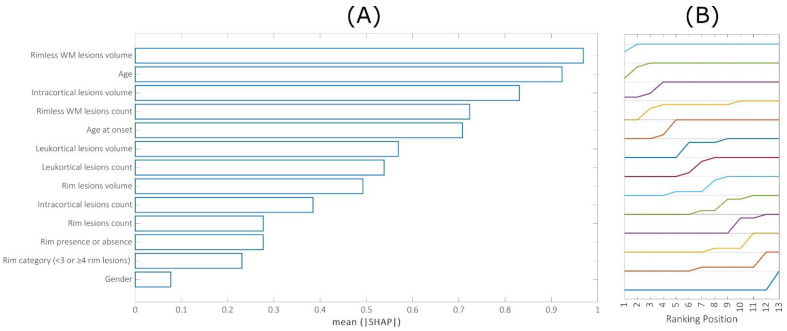
(**A**) feature importance evaluated as the area under the curves shown in (**B**). These curves were obtained by calculating the mean fraction of times (over 50 repetitions) each of the features appeared in each position in single-region rankings.

Figure 6A shows the mean feature importance of 15 regions where significant associations between real and predicted values were found. As a summary measure, we propose the area under the curve, as pictured in Figure 6B. This curve was obtained by calculating the fraction of times (over 50 repetitions) each of the 15 features appeared in each position in single-region rankings, thus generating a global picture based on locally heterogeneous predictions. Interestingly, the features that contributed the most to predictions were the WM lesion volume and count (without considering rim lesions), patient age, and intracortical lesion volume.

To assess the role of different types of demyelinated lesions in the development of cortical atrophy at different disease stages, the analyses above were separately repeated in two groups divided according to disease duration (cutoff: 5 years) by MS (G1 > 5y G2 < 5y). Table 3 shows model performances for the prediction of the average thickness in the whole brain and in the individual hemispheres in both G1 and G2 patient groups.

The prediction performances for G1 were satisfactory (average *p* < 0.002 and r > 0.4), while our models were unable to predict atrophy in G2 (*p* > 0.05 in all cases).

**Figure 7:**
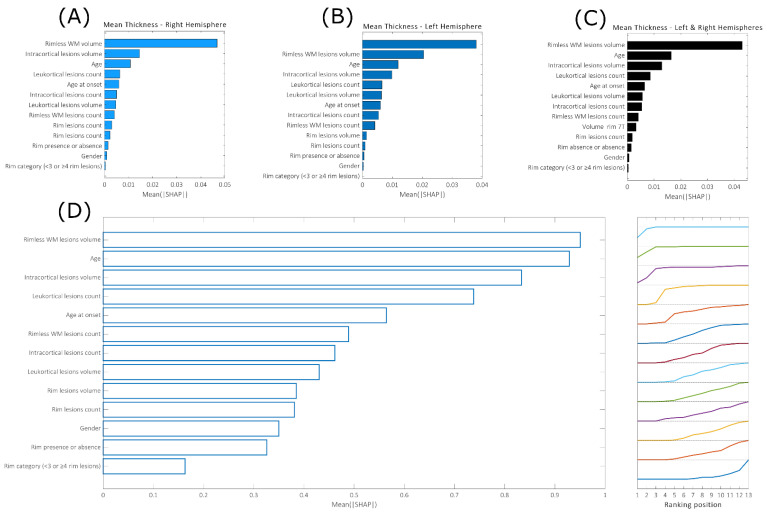
Mean feature importance evaluated across 50 repetitions when predicting thickness in the single hemispheres (**A**,**B**) and in the whole brain (**C**). (**D**): Mean feature ranking position evaluated across 36 brain regions, where the thickness values were assessed in a satisfactory manner (*p* value < 0.05).

Figure 7A–C show the SHAP feature ranking for predicting mean thickness values in patients with disease durations longer than five years (patient group G1). For the prediction in the entire subject population (see Figure 4), the volume of the rimless lesions in WM and the patient’s age also appeared among the main predictors. Both in the whole brain and in individual hemispheres, we found that the volume of the intracortical lesions and the number of leukocortical lesions had a greater importance in the prediction of mean thickness values, suggesting an increased role of cortical lesions in inducing the loss of cortical tissue in patients with a disease duration of 5 years or more. This result was also confirmed when looking at the mean positions in the SHAP feature ranking in the local regions, where the performance of the cortical thickness was satisfactory (*p* value < 0.05, 36 regions in total). In fact, at the local level, thickness values are mainly predicted as (1) rimless WM lesion volumes, (2) patient age, (3) intracortical lesion volume and (4) leukocortical lesion count.

## 4. Discussion

In this study, we developed an interpretable machine learning approach to investigate whether cortical tissue loss in MS is mainly dependent on local pathological processes or disconnection from distant WM lesions and whether lesions that are pathologically known to have a higher degree of tissue destruction [23] could play a critical role in this process. The predictions were formulated in 150 brain regions of 100 MS patients, allowing us to satisfactorily predict cortical thickness values using radiological features mainly extracted from ultrahigh resolution 7T MRI data along with several demographic characteristics. Our results suggest that the most important characteristics in the prediction of cortical thickness are WM lesion load, age, and intracortical lesion volume. Furthermore, we observed that the importance of cortical lesions in predicting cortical tissue loss was greater in patients with a longer duration of disease.

While several previous studies have linked cortical atrophy to WM lesion load [6,7,24], as well as changes in the normal-appearing WM [25,26], other investigations suggest that intracortical demyelination is the most important factor for determining cortical tissue loss [27,28]. However, most of the studies were carried out on scanners with a 1.5 or 3.0 T field strength or used traditional statistical methods with an unknown true prediction power due to their limited generalizability. By combining the increased sensitivity of ultrahigh resolution T2* gradient echo acquisitions at 7.0 T MRI for cortical and rim lesion detection with modern machine learning algorithms to assess the cumulative power and individual importance of cortical and rim lesion types, alongside with traditional imaging markers of disease burden, we demonstrated that both white matter and cortical lesions are the main contributors to cortical tissue loss.

The inclusion of both WM and intracortical lesions in those top-ranking predictors suggests an articulate interaction between close-by pathology and a distanced disconnection.

Interestingly, despite their highly destructive potential, rim lesions per se are not associated with reduced cortical thickness. Previous MRI studies have found, however, that gray matter atrophy, and specifically, the atrophy of deep gray matter structures is more pronounced in patients with rim lesions compared to those without [11,29]. Nevertheless, no study has yet investigated the relationship between rim lesions and cortical tissue loss. The evaluation of the unique, individual contributions of each lesion feature to cortical tissue loss also demonstrated that a small load of the WM lesion is important in the prediction of atrophy. In contrast, the cortical lesion load behaves in the opposite manner, indicating that cortical lesions need to accumulate to produce a similar effect. In fact, GM atrophy is more severe in patients with advanced disease stages [30], the same class of patients in whom histopathological studies have shown the presence of extensive GM demyelination [31,32,33].

Even if we could not predict changes in cortical thickness in early disease stages, we successfully predicted cortical thickness values in the patient group affected by MS for more than five years. Furthermore, our results suggest that, both at the local level and in the whole brain, most of the prediction power is carried by the volume of the rimless WM and intracortical lesions, as a result of the entire population analysis, and by the leukocortical lesion count (more abundant in advanced disease stages [34,35]), demonstrating that depending on the stage of the disease, different types of lesions could play a differential role in the loss of cortical tissue.

At a local level, our model was able to predict thickness values in 15 regions of the brain that belong to both brain hemispheres. Interestingly, the areas where the prediction was successful were the regions most affected by the disease (superior frontal, temporal sulcus, middle temporal, middle temporal gyrus, middle frontal gyrus [36,37], inferior frontal [38], and occipital [37,39] areas). Looking at the mean importance for the prediction in these regions, we again found the same subset of characteristics (WM lesion load, patient age, and intracortical lesion volume) that carry most of the prediction power, pointing to a mechanism that might not be influenced by the region in which it operates.

### Limitations of the Study

The contribution of the intracortical lesion subtype to cortical tissue loss could have been underestimated because even 7 T MRI cannot detect the entire extent of cortical demyelination, although neuropathological–MRI correlations at 7 T have shown that it more than doubles cortical lesion detection in MS compared to lower-field MRI systems [7,8]. In addition, model performances in predicting the mean cortical thickness in all experiments were only fair. Other strategies, e.g., parasitic modeling, should be employed in future research [40]. Thirdly, no model accounted for changes in the cortical normal-appearing gray matter.

## 5. Conclusions

Our results suggest that the overall WM lesion load is the main determinant of cortical tissue loss in MS and is more important than focal cortical demyelination. However, the contribution of cortical lesions to cortical neurodegeneration increases with the duration of the disease. White matter lesion load has a vital effect on cortical thinning when it is low, whereas cortical lesions need to accumulate to produce a similar outcome. Interestingly, WM rim lesions, which are known to be a more aggressive subtype, do not seem to contribute much to overall cortical tissue loss. This might be related to their reduced number, volume and/or slow evolution. Furthermore, rim lesions are not present in all multiple sclerosis patients, while cortical tissue loss is a common finding.

## Figures and Tables

**Table 1:** Mean values and standard deviations (in brackets) of Pearson correlation (r) and corresponding *p* values evaluated between the real and predicted values across 50 repetitions.

Mean Thickness	r-Value	*p* Value
Right Hemisphere	0.47 (0.15)	0.009 (0.0013)
Left Hemisphere	0.44 (0.18)	0.016 (0.020)
Whole Brain	0.48 (0.17)	0.008 (0.011)

**Table 2:** Prediction performances of local cortical thickness values in 15 brain regions belonging to both brain hemispheres where *p* value < 0.05.

LEFT HEMISPHERE			RIGHT HEMISPHERE		
REGION	r Value (SD)	*p*-Pearson (SD)	REGION	r Value (SD)	*p*-Pearson (SD)
Superior frontal gyrus (F1)	0.436 (0.149)	0.016 (0.023)	Superior frontal gyrus (F1)	0.480 (0.125)	0.007 (0.011)
Medial occipitotemporal sulcus (collateral sulcus) and lingual sulcus	0.537 (0.131)	0.002 (0.003)	Medial occipitotemporal sulcus (collateral sulcus) and lingual sulcus	0.539 (0.125)	0.002 (0.003)
Superior temporal sulcus (parallel sulcus)	0.444 (0.121)	0.014 (0.019)	Superior temporal sulcus (parallel sulcus	0.388 (0.212)	0.034 (0.050)
Opercular part of the inferior frontal gyrus	0.420 (0.157)	0.021 (0.030)	Middle-posterior part of the cingulate gyrus and sulcus (pMCC)	0.374 (0.227)	0.042 (0.060)
Long insular gyrus and central sulcus of the insula	0.371 (0.137)	0.044 (0.055)	Middle frontal gyrus (F2)	0.443 (0.178)	0.014 (0.020)
Middle temporal gyrus (T2)	0.396 (0.134)	0.030 (0.042)	Anterior transverse collateral sulcus	0.377 (0.163)	0.040 (0.056)
Medial orbital sulcus (olfactory sulcus)	0.387 (0.104)	0.034 (0.043)	Superior occipital sulcus and transverse occipital sulcus	0.439 (0.116)	0.015 (0.020)
			Lateral occipito-temporal sulcus	0.413 (0.165)	0.023 (0.033)

**Table 3:** Pearson’s correlation (r) coefficients and corresponding *p* values evaluated between the real and predicted values in two different subject groups (G1, with a disease duration > 5 years and G2, disease duration < 5 years).

	G1		G2	
Mean Thickness	r-Value	*p* Value	r-Value	*p* Value
Right Hemisphere	0.46	0.0006	0.19	0.21
Left Hemisphere	0.41	0.002	0.12	0.43
Whole Brain	0.45	0.0007	0.19	0.21

## Data Availability

The sharing of the data depends on Massachusetts General Hospital and Institutional Review Board policy and on the purpose of sharing the data (profit versus nonprofit).
